# Viscosity of concentrated mRNA solutions: Implications for downstream processing

**DOI:** 10.1002/btpr.88529

**Published:** 2026-06-15

**Authors:** Amin Javidanbardan, Fatemeh Najafi, Ziqiao Wang, Andrew L. Zydney

**Affiliations:** ^1^ Department of Chemical Engineering The Pennsylvania State University University Park Pennsylvania USA

**Keywords:** downstream processing, mRNA, saRNA, ultrafiltration, viscosity

## Abstract

Messenger RNA (mRNA) therapeutics can be used to protect against infectious disease and for the treatment of cancers and genetic disorders. In order to obtain the desired therapeutic dosage, it is necessary to concentrate the mRNA to levels where the viscosity of the mRNA solutions can become significant. However, there is currently no quantitative data on the viscosity of these concentrated mRNA solutions. Experiments were performed with two model mRNA constructs: a firefly luciferase (Fluc) mRNA (2.117 kb) and a self‐amplifying RNA (9.442 kb) over a wide range of concentrations. The viscosity of the larger saRNA was more than 140 mPa·s at a concentration of 28 mg/mL, which is more than 10× larger than that for the Fluc mRNA under the same conditions. The viscosity was a weak function of shear rate (from 10 to 100 s^−1^), with some shear‐thinning behavior. The viscosity data were analyzed using several available models from the literature, providing additional insights into the underlying physical behavior. The potential implications of the mRNA viscosity in the downstream processing of mRNA therapeutics are also discussed.

## 1 INTRODUCTION

Messenger RNA (mRNA) has become a cornerstone of modern biotechnology, enabling the rapid development of vaccines and cancer therapeutics.^1^, ^2^ The mRNA is produced by in vitro transcription, followed by a combination of tangential flow filtration (TFF) and chromatography before formulation by encapsulation in lipid nanoparticles (LNP).^3^ The TFF steps provide both concentration of the mRNA and buffer exchange, with the latter also used to remove low molecular weight impurities, including residual nucleotides.^4^

Although not yet investigated for mRNA, it is well‐established that the viscosity can have a dramatic effect on the manufacture and formulation of biologics; this is discussed in some detail in the overview provided by Shire.^5^ The viscosity of concentrated monoclonal antibodies can easily exceed 50 mPa·s at relevant concentrations, although the viscosity can often be controlled by addition of excipients like arginine.^5^ Baek et al.^6^ showed that there was a direct inverse relationship between the maximum achievable protein concentration in TFF and the viscosity of the highly concentrated protein solution based on data for two monoclonal antibodies and an Fc‐fusion protein. This behavior was due to the reduction in protein diffusion coefficient and the increase in the feed‐side pressure drop associated with the increase in the viscosity of the protein solution at high protein concentrations.^7^ Liu et al.^8^ describe several methods for enhancing the performance of TFF for the formulation of highly concentrated monoclonal antibody products, including performing the ultrafiltration at high temperature (46°C) to reduce the viscosity and increase the filtrate flux.

The solution viscosity can also have a large impact on the formulation of mRNA in LNP. At small scales, LNP are commonly made by microfluidic systems,^9^ while larger scale processes typically use impinging jet mixers.^10^ In both cases, the detailed flow/mixing behavior will depend strongly on the viscosity of the mRNA and lipid solutions that are being mixed. Brito et al.^11^ used a combination of planar laser induced fluorescence imaging and computational fluid dynamics to explore how the ratio of the viscosity of the two fluids affects both the balance point and the mixing behavior in confined impinging jet mixers. Pial et al.^12^ showed that the payload heterogeneity in mRNA—LNP formulations is governed by the balance between RNA diffusion kinetics and the dynamics of lipid self‐assembly, with the RNA diffusion coefficient expected to vary inversely with the viscosity of the RNA solution. Hourdel et al.^13^ provided a recent review examining the performance of T‐mixers for producing mRNA‐LNP formulations, highlighting the importance of the Reynolds numbers of the two fluids (which varies inversely with the viscosity).

Littauer and Eisenberg^14^ evaluated the viscosity of dilute solutions of RNA isolated from bacterial protoplasts as a function of both the RNA and NaCl concentrations. Data were analyzed in terms of the reduced viscosity:

(1) $$\eta_{\mathrm{red}}=\frac{\eta_{\mathrm{sp}}}{C}=\frac{1}{C}\left[\frac{\eta }{\eta_{o}}-1\right]$$

where η is the viscosity of the RNA solution, η_sp_ is the specific viscosity, η_o_ is the viscosity of the solvent, and C is the RNA concentration. The reduced viscosity was highest in the absence of salt, with a maximum of 15 mL/mg at an RNA concentration of 0.025 mg/mL. However, data in salt‐free solutions were only obtained up to an RNA concentration of 0.1 mg/mL, with the RNA viscosity being less than 2 mPa·s under these conditions. The viscosity was significantly reduced in the presence of salt, with the maximum value of η_red_ in 1 mM NaCl being less than 2 mL/mg over a range of RNA concentrations.

The viscosity of DNA has been much more widely studied, with the data showing a very large increase in viscosity even at relatively low DNA concentrations. For example, Laesecke and Burger^15^ evaluated the viscosity of calf thymus DNA (with thousands of base pairs) in both capillary and rotating concentric‐cylinder viscometers. The viscosity of a 6 mg/mL solution at room temperature was ~15 mPa·s. The viscosity decreased with increasing temperature between 10 and 50°C but then increased sharply to a maximum value above 50 mPa·s around 70°C, which the authors attributed to the melting of the DNA and the corresponding increase in cross‐linking. In contrast, Elkin et al.^16^ reported viscosities for salmon testes DNA with an average MW of 1300 kDa of about 13,000 mPa·s at a DNA concentration of only 5 mg/mL. Similarly high viscosities (in excess of 1000 mPa·s) were obtained with 6 mg/mL solutions of herring testes and calf thymus DNA.

Despite the importance of the viscosity of mRNA solutions in both TFF and LNP formation, there is currently an absence of quantitative data on the viscosity of well‐defined purified RNA molecules as a function of the RNA concentration. This study aims to fill this gap by evaluating the viscosity of two clinically relevant mRNA constructs, a 2117 nucleotide (nt) mRNA (Fluc) and a large 9442 nt self‐amplifying RNA (saRNA), over the range of concentrations relevant to industrial processes. The viscosity data were analyzed using a number of available models for the viscosity of concentrated polyelectrolytes, providing additional insights into the factors controlling the mRNA viscosity.

## 2 MATERIALS AND METHODS

### 2.1 Materials

Purified solutions of Firefly luciferase (Fluc) mRNA (~2117 nt, corresponding to a molecular weight of ~700 kDa) and a self‐amplifying RNA (saRNA, 9442 nt and ~ 3100 kDa) were provided by Arranta Bio (Boxborough, MA). The mRNA constructs were synthesized from the appropriate DNA templates by in vitro transcription and purified by TFF and oligo‐dT affinity chromatography.^17^ The GC content of the Fluc mRNA was ~60% while that for the saRNA was 50%. The RNA was exchanged into deionized water and concentrated to the desired final concentration using 30 kDa Amicon® Ultra centrifugal filters (15, 4, and 0.5 mL capacities based on the target concentration factor). Small‐volume devices were centrifuged using an Eppendorf 5425 microcentrifuge, whereas larger‐volume devices were processed in an Eppendorf 5810 refrigerated centrifuge operated at 21°C. The applied relative centrifugal force (RCF) was set to 50% of the manufacturer's recommended maximum. Retentate samples were subsequently collected for mRNA quantification using Qubit™ RNA XR (Extended Range) Assay Kits in conjunction with a Qubit® Flex Fluorometer (Thermo Fisher Scientific) following the manufacturer's instructions.

### 2.2 Viscosity measurement

Viscosity measurements were performed using an EMS‐1000S Viscometer (Kyoto Electronics Manufacturing Co., Ltd., Kyoto, Japan). This instrument employs an electromagnetic spinning (EMS) method, which is particularly well‐suited for analysis of biological samples since it requires small sample volumes (~90 μL) and can evaluate the viscosity from 0.1 to 10^5^ mPa·s with high precision. The EMS Viscometer uses a suspended aluminum alloy sphere (1.5 mm) that is spun with an electromagnetic field, with the viscosity determined from the sphere's rotational speed in response to a defined torque.

Experiments were conducted over a wide range of RNA concentrations up to near‐gelation conditions: 0.5–70 mg/mL for Fluc mRNA and 0.1–28 mg/mL for saRNA. Measurements were performed at multiple rotation speeds (500–1000 rpm) to assess the effect of shear rate on the measured viscosity. All measurements were conducted at pH ≈ 7 and at ambient temperature (~25°C) with DI water used as the reference solvent (η_o_ = 1.0 mPa·s). The viscosity of mRNA should be relatively independent of solution pH (between pH 4 and 7) since the pK_a_ of the phosphate groups is between pH 1 and 2. This is consistent with literature data for DNA showing constant viscosity below pH 8.^18^

### 2.3 RNA characterization

The hydrodynamic radii of the mRNA constructs were evaluated by dynamic light scattering (DLS) using a Malvern Zetasizer Nano ZS90 (Malvern, UK). Each sample was illuminated with a 632.8 nm laser, with the scattered light intensity at 90° evaluated using an avalanche photodiode detector. Ten repeat measurements were obtained, each with a 30 s acquisition time. The hydrodynamic radius was evaluated from the measured diffusion coefficient using the Stokes‐Einstein equation.

## 3 RESULTS AND DISCUSSION

### 3.1 Shear rate effects

Figure 1 shows the effect of shear rate on the viscosity of the Fluc mRNA and saRNA at concentrations of 5 and 20 mg/mL. The viscosity of the 5 mg/mL solutions was nearly independent of shear rate, decreasing by less than 5% over more than a twofold range of shear rate. The viscosity for the 20 mg/mL solutions shows a somewhat stronger dependence on the shear rate, with the viscosity decreasing by 10% as the shear rate increases. The average values of the viscosity for the 5 and 20 mg/mL concentrations of the Fluc mRNA were 3.9 and 7.2 mPa·s, respectively. The viscosity of the 20 mg/mL solution of the saRNA is more than 73 mPa·s over the full range of shear rates, which is 10× the viscosity of the Fluc mRNA at the same concentration, consistent with the much longer length of the saRNA (9442 vs. 2117 nt). There was no evidence of RNA degradation in any of the experiments, with DLS data obtained after the viscosity measurements showing statistically identical size distributions as those evaluated before the experiment.

**FIGURE 1:**
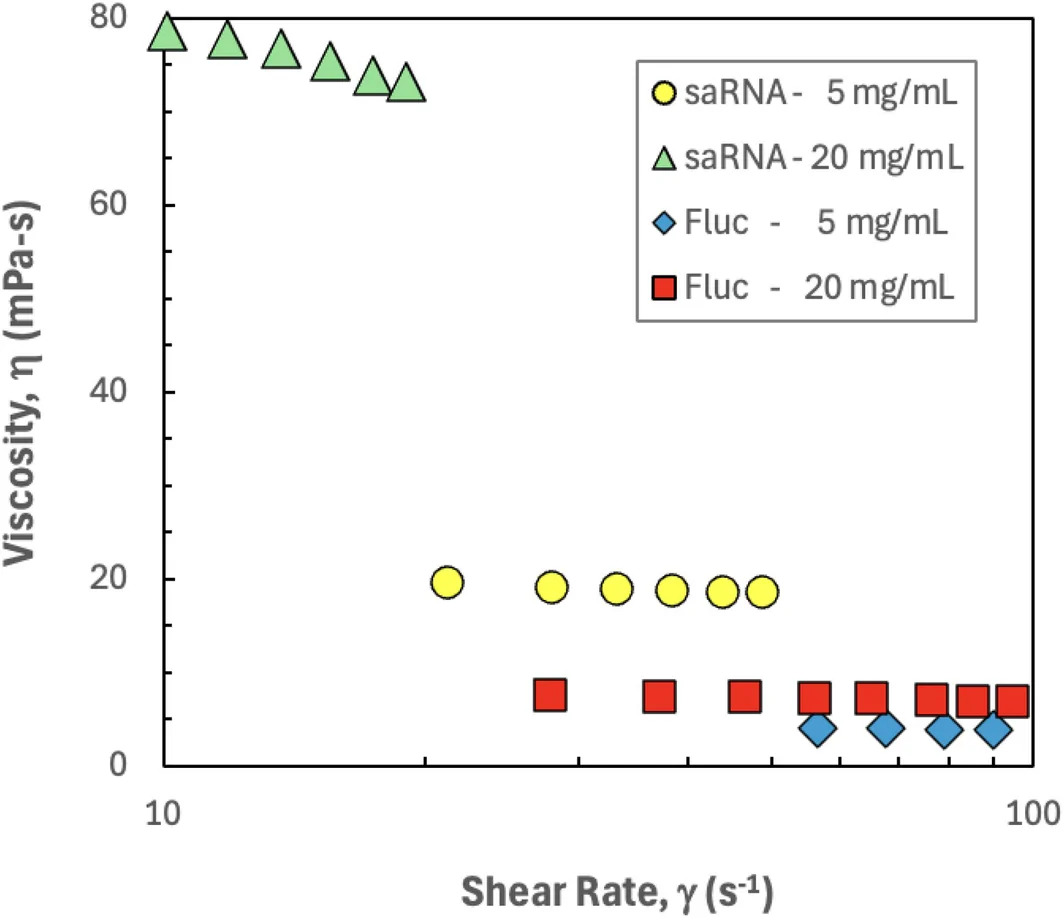
Viscosity of solutions of the Fluc mRNA (2117 nt) and saRNA (9442 nt) in DI water at concentrations of 5 and 20 mg/mL as a function of the average shear rate in the EMS‐1000S Viscometer.

There are no literature data on the shear rate dependence of the viscosity of mRNA. However, a somewhat greater degree of shear‐thinning was observed by Eisenberg^19^ in relatively dilute solutions of herring sperm DNA with an average MW = 6300 kDa. For example, the solution viscosity at a DNA concentration of 0.052 mg/mL in the presence of 1 mM NaCl decreased by about one‐third as the shear rate increased from 1 to 17 s^−1^, with the degree of shear thinning decreasing in the presence of higher NaCl concentrations. However, it is impossible to extrapolate from these results to the behavior of the RNA constructs examined in this work given the much more rigid structure (longer persistence length) of the double‐stranded DNA.

### 3.2 Concentration dependence

The effect of the RNA concentration on the viscosity of both the Fluc mRNA and saRNA is examined explicitly in Figure 2. The data were all obtained at a programmed motor rotation of 600 rpm; this corresponds to a range of probe rotation rates (and thus a range of shear rates) based on the viscosity of the mRNA solution. The viscosity of the saRNA was uniformly larger than that of the Fluc mRNA, with this difference increasing with increasing concentration. For example, the viscosity of the saRNA at a concentration of 5 mg/mL was ~5× that for the Fluc mRNA, but this increased to more than 10x at a concentration of 20 mg/mL. The viscosity of the Fluc mRNA increased from 2.1 mPa·s at an RNA concentration of 0.5 mg/mL to 45 mPa·s at 70 mg/mL, corresponding to a 21‐fold increase over this concentration range. The viscosity remained below 10 mPa·s up to a concentration of 30 mg/mL but then increased to more than 45 mPa·s at a concentration of 70 mg/mL. The viscosity of the saRNA was much larger than that for the Fluc mRNA and showed a much stronger dependence on the RNA concentration. For example, the viscosity increases from 1.6 mPa·s at 0.1 mg/mL to 150 mPa·s at 28 mg/mL, a 90‐fold increase over an even smaller concentration range than that explored for the Fluc mRNA. The viscosity of the saRNA solution exceeded 50 mPa·s at a concentration of only 15 mg/mL and increased to more than 140 mPa·s at a concentration of 28 mg/mL.

**FIGURE 2:**
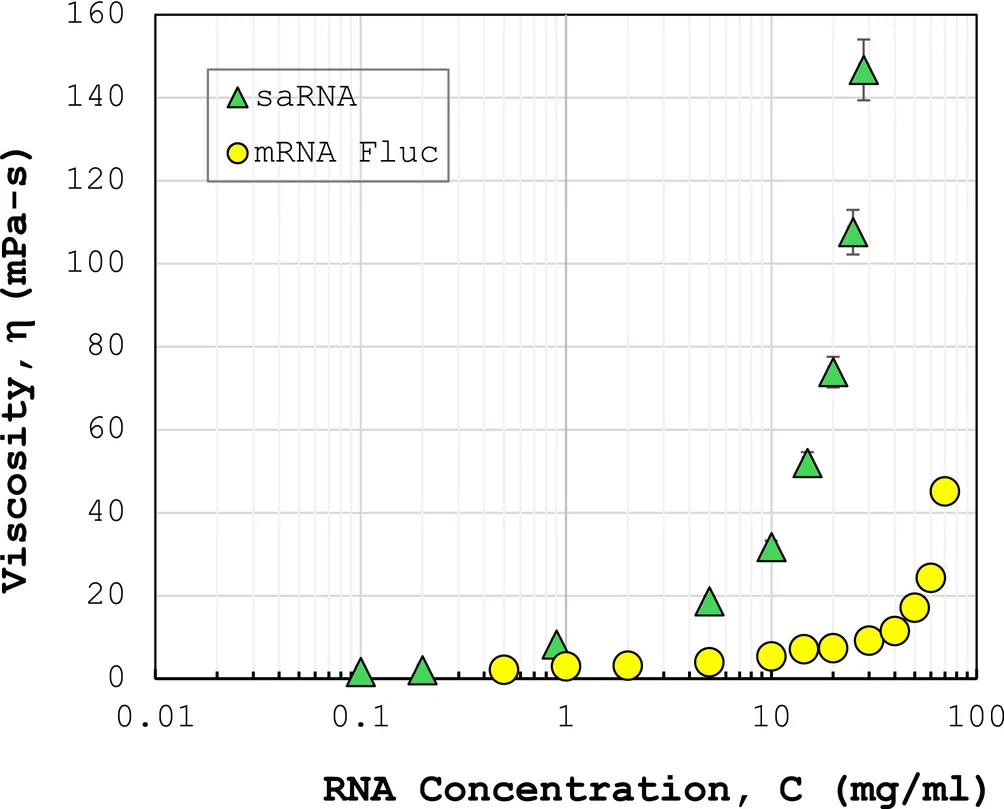
Viscosity of saRNA (9442 nt) and mRNA Fluc (2117 nt) solutions in ultrapure DI water as a function of RNA concentration. Data obtained in the ESM‐1000S Viscometer at a rotation speed of 600 rpm.

Although there are no literature data for the viscosity of mRNA at the concentrations examined in this work, there are limited data for the viscosity of DNA under comparable conditions that can provide additional insights into the factors controlling the viscosity of the nucleic acid solutions. Figure 3 compares our results for the viscosity of Fluc mRNA and saRNA with data obtained by Elkin et al.^16^ for the viscosity of salmon testes DNA with an average MW = 1300 kDa (between the MW of the Fluc mRNA and the saRNA). The viscosity of the DNA solution at pH 6.4 and a concentration of 4 mg/mL was 1910 mPa·s, which is more than two orders of magnitude larger than that for the RNA samples, due to the much more rigid structure of the double‐stranded DNA. The DNA viscosity decreased to 19 mPa·s at pH 4 where the DNA is present in a denatured, single‐stranded, form that is closer to the structure of RNA. The differences in viscosity of the mRNA and DNA are likely due to differences in the extent of intramolecular base pairing as well as differences in the size distribution of the nucleic acids; the Fluc mRNA is a pure sample while the salmon testes DNA consists of a wide range of DNA sequences and sizes. Further studies would be required to obtain a more quantitative understanding of this phenomenon.

**FIGURE 3:**
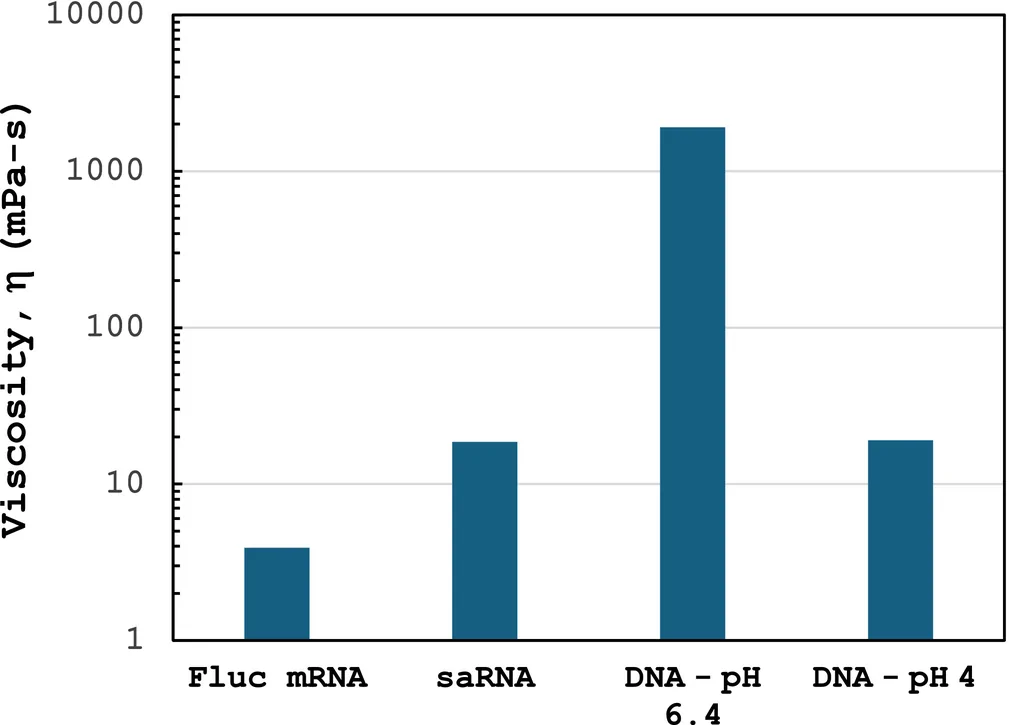
Comparison of viscosity for Fluc mRNA and saRNA (both at 5 mg/mL) with that of a 1300 kDa average molecular weight DNA (at 4 mg/mL). Data for DNA with ~2000 base pairs obtained from Elkin et al.^16^ Data for saRNA (9442 nt) and mRNA Fluc (2117 nt) obtained in this study.

### 3.3 mRNA characterization

One possible cause for the increase in viscosity at high RNA concentrations could be the formation of RNA clusters due to self‐association/aggregation; this phenomenon is well known to influence the viscosity of many protein solutions.^20^, ^21^ The extent of RNA association was examined by dynamic light scattering (DLS), with typical results for the volume‐weighted size distributions for the Fluc mRNA (top panel) and saRNA (bottom panel) at 0.5 and 12 mg/mL shown in Figure 4. All samples are monodisperse, with volume‐weighted average hydrodynamic diameters of 28 and 9.4 nm for the Fluc mRNA (at 0.5 and 12 mg/mL) and 63 and 23 nm for the saRNA. In both cases, the effective hydrodynamic diameter at 12 mg/mL was approximately three times smaller than that in the dilute solution. In addition, there was no evidence of any saRNA aggregation; given the strong dependence of the scattering intensity on particle size, DLS should be able to detect the presence of large saRNA aggregates even at concentrations well below 1% by mass.^22^

**FIGURE 4:**
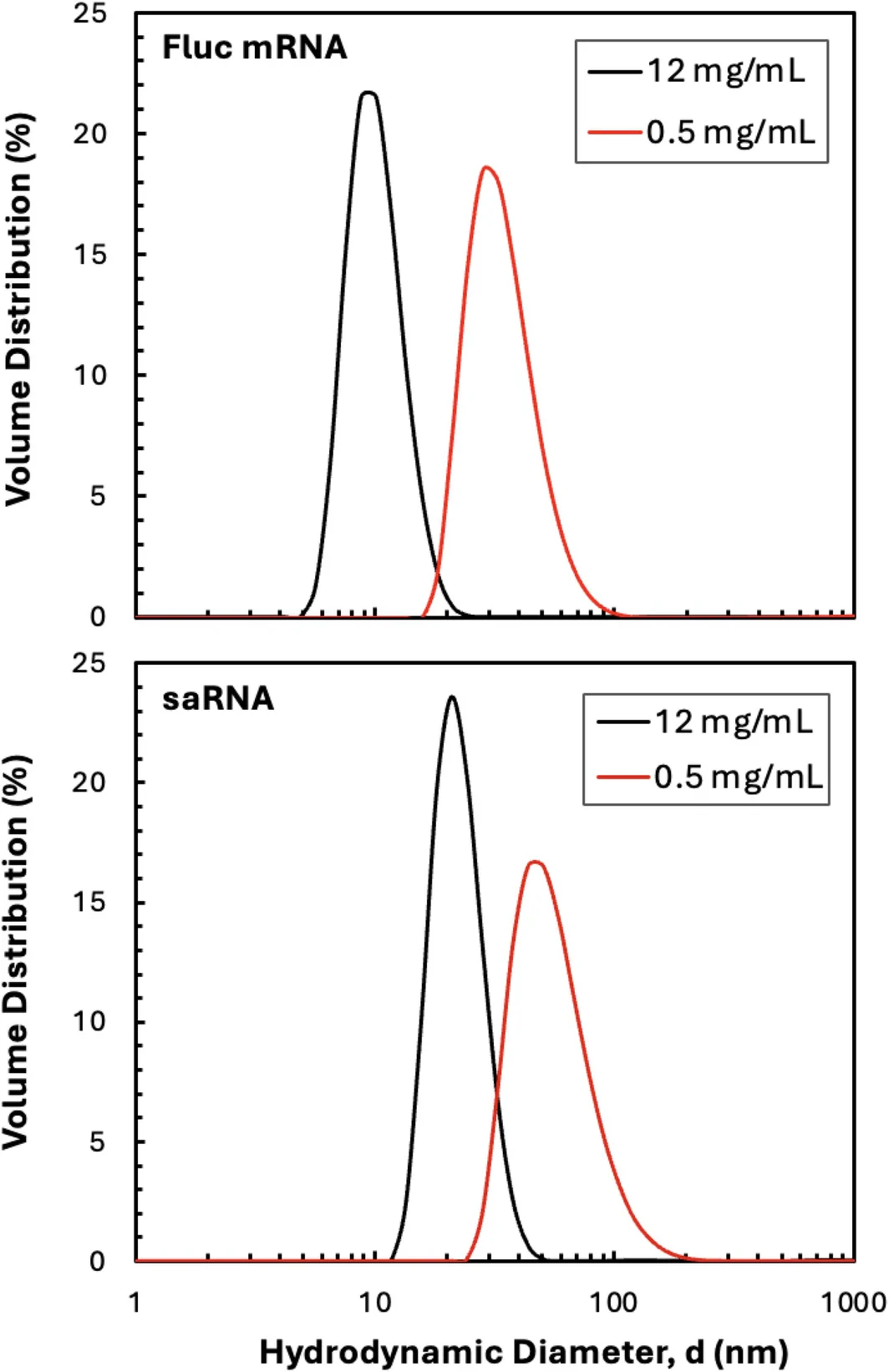
Volume‐weighted size distributions obtained using dynamic light scattering for the Fluc mRNA (2117 nt—top panel) and the saRNA (9442 nt—bottom panel) at concentrations of 0.5 and 12 mg/mL in ultrapure DI water.

The effect of the RNA concentration on the measured diffusion coefficients (inversely proportional to the effective hydrodynamic diameter) is shown explicitly in Figure 5. The diffusion coefficient of both the Fluc mRNA and the saRNA initially increases with increasing concentration, consistent with a positive value of the diffusion interaction parameter, k_D_:

(2) $$D=D_{o}\left[1+k_{D}C\right]$$

where D_o_ is the diffusion coefficient at infinite dilution. The saRNA diffusion coefficient becomes independent of the saRNA concentration at C > 5 mg/mL with a value of ~22 μm^2^/s, with the diffusion coefficient of the smaller Fluc mRNA approaching a value of 55 μm^2^/s above a concentration of 10 mg/mL. Similar behavior has been reported for the fast diffusion coefficient of polyelectrolytes, including data obtained by Sedlak^23^ for salt‐free solutions of sodium poly(styrenesulfonate) and by Forster et al.^24^ for quaternized poly(2‐vinylpyridine), although in both cases the plateau was obtained at polymer concentrations above about 0.1 mg/mL. The plateau is consistent with the behavior of semi‐dilute polyelectrolyte solutions in which the diffusivity is independent of both the concentration and molecular weight of the polymer, with the motion of the polyelectrolyte described by the gel mode associated with the transient network arising from cooperative fluctuations^25^.

**FIGURE 5:**
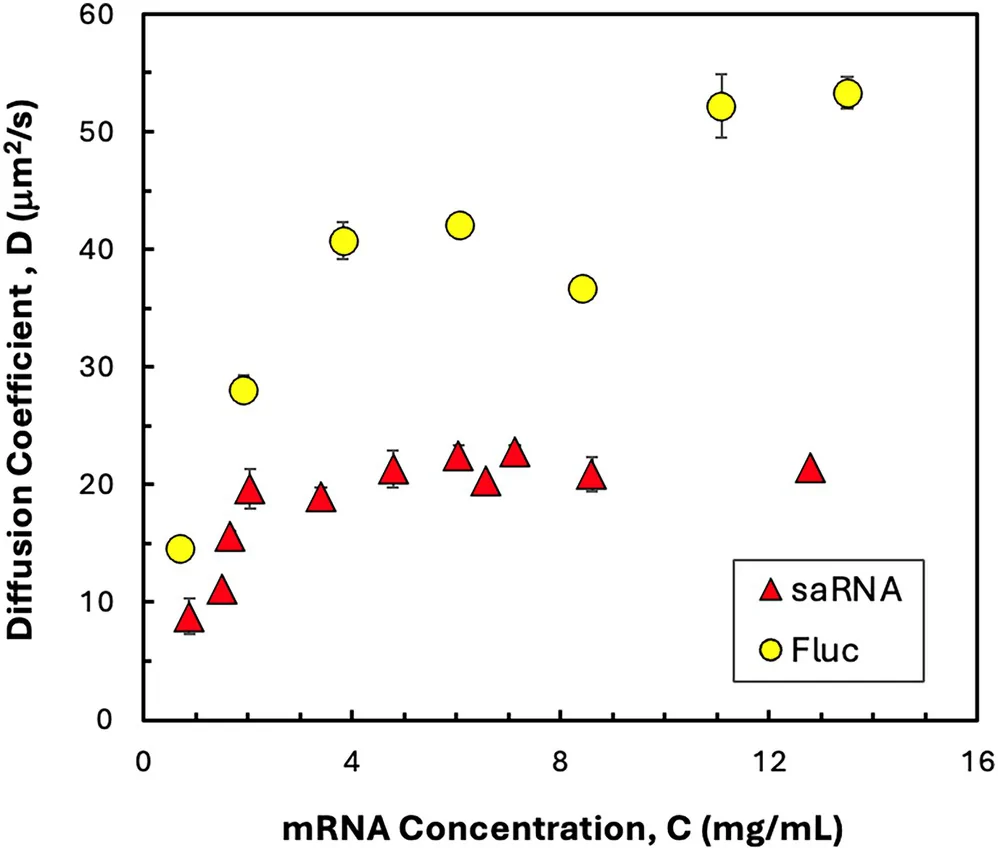
Diffusion coefficients of the Fluc mRNA (2117 nt) and the saRNA (9442 nt) evaluated using dynamic light scattering as a function of the saRNA concentration in ultrapure DI water.

### 3.4 Comparison with viscosity models

A number of previous studies of polymer and DNA viscosity have analyzed the viscosity data using the Huggins model^26^:

(3) $$\eta_{\mathrm{red}}=\left[\eta \right]+k_{H}{\left[\eta \right]}^{2}C$$

where [η] is the intrinsic viscosity and k_H_ is the Huggins constant. The data in Figure 2 have been replotted in Figure 6 as the reduced viscosity as a function of the RNA concentration. The intrinsic viscosity, evaluated as the y‐axis intercept, was about 2.3 mL/mg for the Fluc mRNA and around 8 mL/mg for the saRNA. This difference is qualitatively consistent with the difference in MW of the two RNA constructs. In both cases, the reduced viscosity initially decreases with increasing RNA concentration, going through a minimum at C ≈ 10 mg/mL for the saRNA and C ≈ 40 mg/mL for the Fluc mRNA. The presence of a minimum in the plot of η_red_ versus concentration cannot be predicted by the classical form of the Huggins model (Equation 3). In sharp contrast, data obtained by Alvarez et al.^27^ showed a linear increase in reduced viscosity for DNA with increasing concentration (up to DNA concentrations of 4 mg/mL), which is in good agreement with Equation (3), again highlighting the large difference in rheological behavior of single‐stranded RNA and double‐stranded DNA.

**FIGURE 6:**
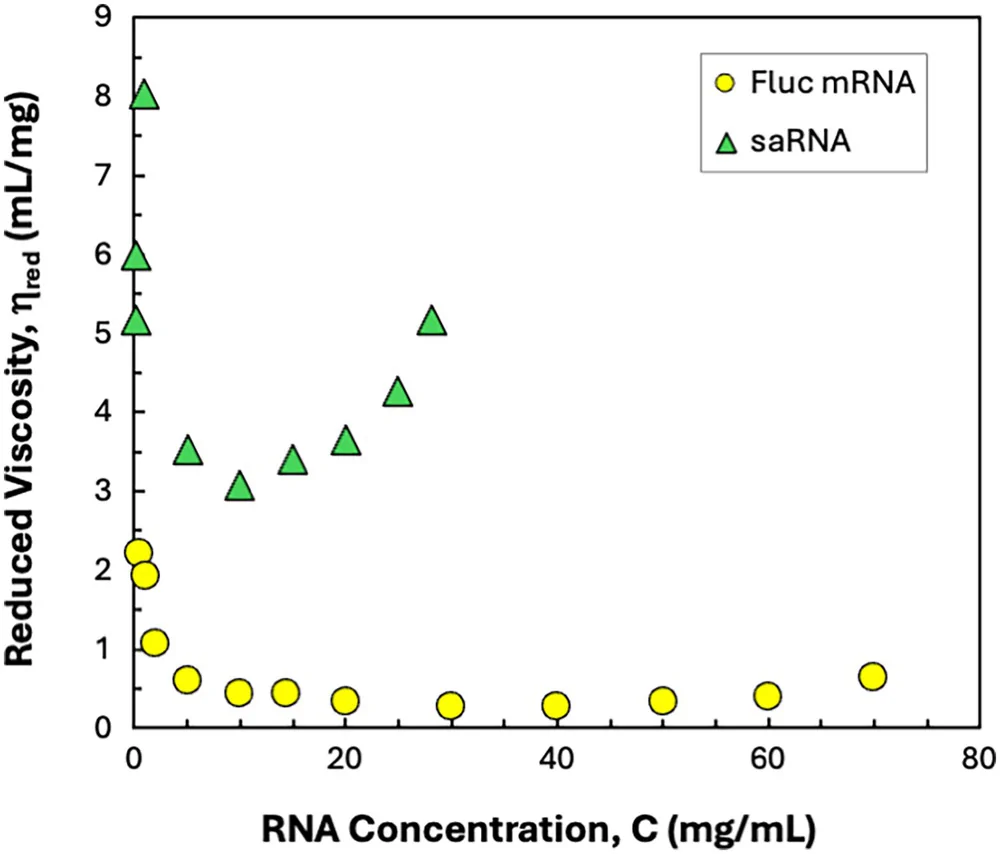
Reduced viscosity of Fluc mRNA (2117 nt) and saRNA (9442 nt) solutions as a function of RNA concentration. Data obtained in the ESM‐1000S Viscometer at a rotation speed of 600 rpm.

Recent work by Gulati and Lopez^28^ found that the specific viscosity of concentrated polyelectrolytes could be well‐represented by a modification of Fuoss' law^29^:

(4) $$\eta_{\mathrm{sp}}={\left(\frac{C}{C^{*}}\right)}^{1/2}\exp\left(\mathrm{AC}\right)$$

where C* is the overlap concentration (the development by Gulati and Lopez used the polymer volume fraction instead of the concentration in the exponential). The original equation due to Fuoss neglects the exponential term. Equation (4) predicts a minimum in the reduced viscosity at C = ½ A^−1^, consistent with the results in Figure 6. Figure 7 shows a comparison between the viscosity data and the model correlations given by Equation (4) using the best fit values of A and C* determined by linear regression by plotting the logarithm of $\eta_{\mathrm{sp}}C^{-1/2}$ as a function of C. The model correlations are in good agreement with the experimental results, with the mean sum of the squared residuals less than 7.5% for both constructs, providing simple mathematical expressions that can be used to estimate the viscosity as a function of the RNA concentration.

**FIGURE 7:**
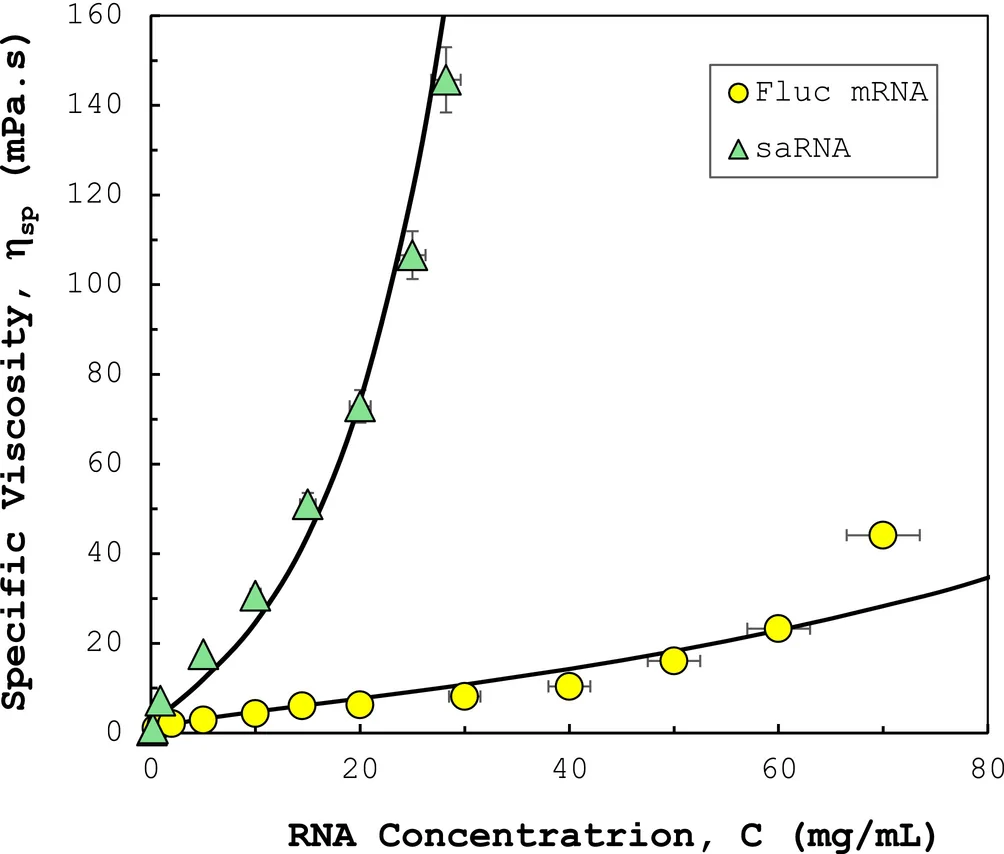
Specific viscosity of Fluc mRNA (2117 nt) and saRNA (9442 nt) solutions as a function of RNA concentration. Solid curves are model fits given by Equation (4).

The best fit values of C* and A determined from the linear regression fits are summarized in Table 1. The overlap concentration for the Fluc mRNA is much larger than that for the saRNA, as expected. Scaling arguments suggest that C* for long linear polyelectrolytes should scale with the molecular weight as^30^:

(5) $$C^{*}\approx M^{-2}$$

which predicts a ratio of $C_{\text{Fluc}}^{*}/C_{\text{saRNA}}^{*}$ = 20 compared to the ratio of the best fit values of 7.8. This difference may reflect the different degree of intramolecular interactions related to regions of internal complementarity in the two RNA constructs. The exponential parameter for saRNA is about five times larger than that for the Fluc mRNA, consistent with the much higher value of the RNA concentration giving the minimum value of $\eta_{\mathrm{red}}$ seen in Figure 6.

**TABLE 1:** Best fit values and standard errors of the model parameters in Equation (4) determined from the viscosity data for Fluc mRNA and saRNA.

	Fluc mRNA	saRNA
C* (mg/mL)	0.58 ± 0.12	0.074 ± 0.033
A (mL/mg)	0.013 ± 0.003	0.076 ± 0.014
$\frac{1}{N}\sum_{i}^{N}{\left(1-\frac{\eta_{\text{pred}}}{\eta_{\exp}}\right)}^{2}$	0.051	0.073

## 4 DISCUSSION

mRNA therapeutics are typically encapsulated in LNP to protect the mRNA from enzymatic degradation and to facilitate cellular uptake. The LNP are formed by combining a lipid mixture (in ethanol) with an aqueous phase containing the mRNA at a concentration between 0.1 and 1.0 mg/mL. Even though these seem like very dilute solutions, the viscosity of the Fluc mRNA was 2.9 mPa·s at a concentration of 1 mg/mL while the viscosity of the much longer saRNA was greater than 8 mPa·s compared to the viscosity of the lipids in the ethanol solution, which is typically assumed to be close to that of pure ethanol (~1.2 mPa·s). Thus, there can be a large difference in the viscosity between the two phases that need to be mixed, with the magnitude of this difference being a strong function of the length of the mRNA construct. As discussed in the Introduction, this viscosity ratio can have a significant impact on LNP formulation, an effect that has been largely unappreciated in the literature on LNP formulation.

More significantly, a number of studies have discussed the potential for using much higher mRNA concentrations in the development of mRNA therapeutics. For example, the patent application by Karve et al.^31^ specifically describes mixing a suitable lipid solution with a solution containing “mRNA at a concentration up to about 5.0 mg/mL,” with this mRNA solution formed by diluting a stock solution with an mRNA concentration of “about 100 mg/mL,” although no data were provided under these conditions. The development of an effective mixing process using these highly concentrated mRNA solutions would be critically affected by the viscosity of the mRNA; even at 5 mg/mL, the saRNA construct examined in this work had a viscosity of 18.6 mPa·s.

In addition, the high viscosity of the concentrated mRNA solution will have a significant impact on the use of TFF for concentration and buffer exchange in the downstream processing of the mRNA. Cui et al.^32^ specifically noted a decay in filtrate flux when the mRNA concentration exceeded 1.5 mg/mL, likely due to an increase in membrane fouling. Concentration polarization effects in TFF will result in mRNA concentrations at the membrane surface that could be as much as 10 or 100× larger than that in the feed, with the high viscosity of this highly concentrated region having a significant impact on the performance of the TFF process.^33^ Considerable work has been done in the area of protein ultrafiltration to develop TFF systems that can handle the high viscosity of concentrated protein solutions,^34^ including the development of specialized cassettes with lower feed channel resistance.^35^ Future work will be required to determine the potential benefits of applying these strategies for processing more highly concentrated solutions of mRNA.

It is also possible that the chromatographic steps used in the purification of mRNA therapeutics will be adversely affected by the viscosity of more concentrated mRNA solutions encountered in intensified mRNA processes. The impact of solution viscosity of preparative chromatography of food proteins is discussed in detail by Schultze‐Jena et al.^36^ Increasing viscosity leads to greater pressure drop through the column and reduced mass transfer rates in both the bulk solution and resin pores, and it can also affect column dispersion due to viscous fingering.^37^ The viscosity data obtained in this work should help inform the design and appropriate operation of the oligo‐DT and ion exchange chromatography steps commonly used in the downstream processing of mRNA.

## 5 CONCLUSIONS

This study provides the first experimental data for the viscosity of highly purified mRNA solutions at high concentrations using two mRNA constructs with very different length. The RNA viscosity is much smaller than that of double‐stranded DNA, reflecting the much greater flexibility of the single‐stranded RNA. The viscosity of the saRNA (9442 nucleotides) was considerably larger than that for Fluc mRNA (2117 nucleotides), particularly at high concentrations, demonstrating the importance of the length of the RNA construct. The viscosity data were well described using a recently developed model by Gulati and Lopez,^28^ including the presence of a distinct minimum in the plot of the reduced viscosity as a function of the RNA concentration. These results have important implications for understanding the behavior of concentrated RNA solutions and for the design of downstream processes for the manufacture of RNA therapeutics.

## AUTHOR CONTRIBUTIONS

**Andrew L. Zydney:** Conceptualization; supervision; funding acquisition; project administration; writing – review and editing. **Amin Javidanbardan:** Conceptualization; methodology; investigation; formal analysis; writing – original draft. **Fatemeh Najafi:** Investigation; formal analysis; visualization. **Ziqiao Wang:** Investigation; visualization; formal analysis.

## CONFLICT OF INTEREST STATEMENT

The authors declare no conflicts of interest.

## DISCLAIMER

This report was prepared as an account of work sponsored by an agency of the United States Government. Neither the United States Government nor any agency thereof, nor any of their employees, makes any warranty, express or implied, or assumes any legal liability or responsibility for the accuracy, completeness, or usefulness of any information, apparatus, product, or process disclosed, or represents that its use would not infringe privately owned rights. Reference herein to any specific commercial product, process, or service by trade name, trademark, manufacturer, or otherwise does not necessarily constitute or imply its endorsement, recommendation, or favoring by the United States Government or any agency thereof. The views and opinions of authors expressed herein do not necessarily state or reflect those of the United States Government or any agency thereof.

## Supporting information


**Data S1:** Supporting Information.

## Data Availability

The experimental data are provided in the Supporting Information Excel file.
